# Two New Jaspamide Derivatives from the Marine Sponge *Jaspis splendens*

**DOI:** 10.3390/md7030435

**Published:** 2009-09-15

**Authors:** Sherif S. Ebada, Victor Wray, Nicole J. de Voogd, Zhiwei Deng, Wenhan Lin, Peter Proksch

**Affiliations:** 1 Institute of Pharmaceutical Biology and Biotechnology, Heinrich-Heine University, Universitaetsstrasse 1, D-40225 Duesseldorf, Germany; E-Mail:sherif.Elsayed@uni-duesseldorf.de (S.S.E.); 2 Helmholtz Center for Infection Research, Inhoffenstrasse 7, D-38124 Braunschweig, Germany; E-Mail:victor.wray@helmholtz-hzi.de (V.W.); 3 National Museum of Natural History, PO Box 9517 2300 RA Leiden, The Netherlands; E-Mail:voogd@naturalis.nnm.nl (N.J.V.); 4 Analytical and Testing Center, Beijing Normal University, Beijing 100875, China; E-Mail:dengzw@bnu.edu.cn (Z.W.D.); 5 State Key Laboratory of Natural and Biomimetic Drugs, Peking University, Beijing 100083, China; 6 Department of Pharmacognosy and Phytochemistry, Faculty of Pharmacy, Ain-Shams University, Abbasia, Cairo, Egypt; E-Mail:sss3ebada@yahoo.com (S.S.E.)

**Keywords:** Jaspis splendens, jaspamide Q and R, structure elucidation, cytotoxic activity

## Abstract

Two new jaspamide derivatives **2** and **3**, together with the parent compound jaspamide (**1**) have been isolated from the marine sponge *Jaspis splendens* collected in Kalimantan (Indonesia). The structures of the new compounds were unambiguously elucidated based on 1D and 2D NMR spectral data, mass spectrometry and comparison with jaspamide (**1**). The new derivatives inhibited the growth of mouse lymphoma (L5178Y) cell line *in vitro* with IC_50_ values of <0.1 *μ*g/mL.

## 1. Introduction

Peptides are well-known bioactive metabolites from marine invertebrates [1,2] including linear peptides [3,4], depsipeptides [5,6], cyclic [7–11] and bicyclic peptides [12,13]. Sponges of the genus *Jaspis* (family, Jaspidae) have been a rich source of biologically active, structurally novel natural products. After the discovery of the cyclodepsipeptide jaspamide (jasplakinolide, **1**) in the sponge *Jaspis* cf. *johnstoni* in 1986 by Ireland [14] and Crews [15], which is known for its pronounced biological activities including antifungal [16], anthelmintic, insecticidal [14,15] and cytotoxic activity [17], sponges of the genus *Jaspis* have received considerable attention, and since then the chemistry of *Jaspis* sponges has been the subject of more than 90 publications. A wide variety of constituents has been isolated from this genus including several jaspamide derivatives from *Jaspis splendens* [18–21], isomalabaricane triterpenes from *Jaspis stellifera* [22–24] and other species [25–29], cytotoxic macrolides from the Okinawan sponge *Jaspis* sp. [30], bengazoles [31,32] that stand out as unique bis-oxazoles containing a carbohydrate-like polyol side chain, antiparasitic, antimicrobial, and cytotoxic amino acid derivatives known as bengamides [33–35], cytotoxic bromotyrosine derivatives [36,37], and a series of dihydroxystyrene sulphate derivatives [38–42].

**Figure 1:**
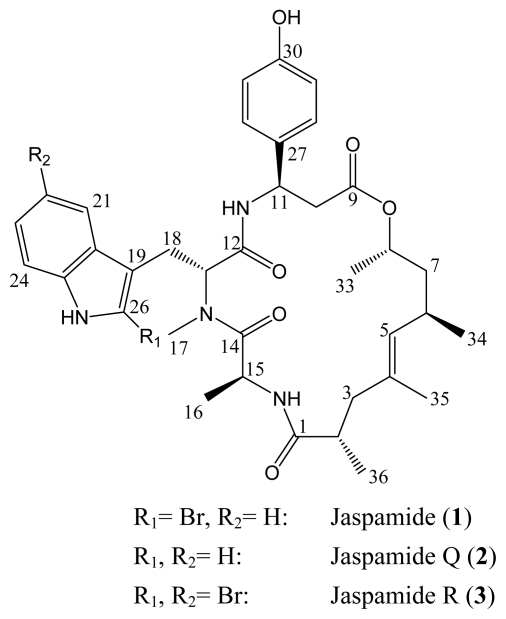
New jaspamide derivatives **2** and **3**, together with the parent jaspamide (**1**).

As part of our ongoing studies on bioactive natural products from marine sponges, we investigated a specimen of *Jaspis splendens* collected in Kalimantan (Indonesia). The crude methanolic extract exhibited considerable *in vitro* cytotoxic activity against mouse lymphoma L5178Y cells. Chromatographic separation of the extract yielded jaspamide (**1**) as the major constituent. Through bioactivity-guided chemical investigation of the ethyl acetate soluble fraction minor analogues of jaspamide, including the new natural products jaspamide Q (**2**), and jaspamide R (**3**) (Figure 1) were obtained. In this paper, we describe isolation, structural elucidation, and biological activity of the new jaspamide derivatives, both of which carry a modified 2-bromoabrine (*N*-methyltryptophan) residue compared to jaspamide (**1**).

## 2. Results and Discussion

Total methanolic extract of the sponge *J. splendens* was partitioned according to the scheme previously described by Ebada *et al*. [43]. The bioactive ethyl acetate soluble fraction was further chromatographed by Sephadex LH20 column chromatography followed by reversed-phase preparative HPLC to give jaspamide (**1**, 0.0013%, dry weight), jaspamide Q (**2**, 0.00001%, dry weight), and jaspamide R (**3**, 0.00001%, dry weight).

Jaspamide Q (**2**) was obtained as a white amorphous solid, and the ESIMS spectrum showed a pseudomolecular ion peak at *m/z* 631.3 [M+H]^+^, which was 79 amu smaller than that of jaspamide (**1**), the parent compound. This difference was assigned to the absence of the bromine atom at C-26 in jaspamide (**1**). The molecular formula of jaspamide Q (**2**) was C_36_H_46_N_4_O_6_, based on HRFTMS (*m/z* 631.3491 [M+H]^+^, Δ + 1.0 ppm), therefore jaspamide Q (**2**) was identified as the debromo analogue of **1**. ^1^H-NMR spectral data (Table 1) revealed that the resonances of **2** were superimposable with those of **1** with only one additional proton resonance at δ_H_ 6.87 (1H, br *s*) that was ascribed to H-26. The complete structure of jaspamide Q (**2**) was unambiguously elucidated and assigned on the basis of ^1^H–^1^H COSY, TOCSY, ROESY, and HMBC spectra.

In particular, the similarity of ^1^H-, and ^13^C-NMR resonances between jaspamide Q (**2**) and jaspamide (**1**) implied that the chiral centers of alanine, abrine (*N*-methyltryptophan), *β*-tyrosine, and of the polypropionate fragment had the same relative configurations in both molecules. Therefore, the stereochemistry depicted in Figure 1 was tentatively assigned by analogy with the parent compound together with ROESY spectra that revealed a clear correlation between Me-16 and Me-34; and Me-33 and Me-36. However, an apparent deshielding of Me-16 was noted in **2 [**δ_H_ 1.04 (3H, *d*, 6.6 Hz)] compared to **1 [**δ_H_ 0.70 (3H, *d*, 6.7 Hz)] resembling that between jaspamide M [δ_H_ 1.17 (3H, *d*, 6.8 Hz)] [21] and jaspamide H [δ_H_ 0.72 (3H, *d*, 6.7 Hz)] [20]. These differences in chemical shifts for the latter congeners were proven to be caused by d-Ala or l-Ala residues, respectively. Anaylsis of the absolute configurations of the amino acids of **2** could not be performed due to the small amount of compound isolated (0.7 mg).

**Figure 2:**
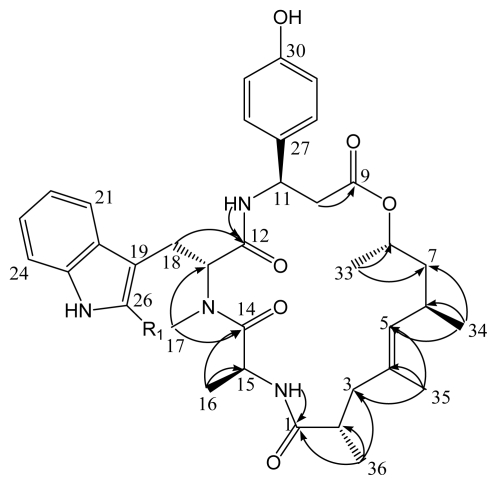
Key HMBC correlations of jaspamide Q (**2**).

A partial ^13^C-NMR assignment of jaspamide Q (**2**) was achieved through HMBC spectra (Figure 2) which revealed clear correlations at δ_c_ 174.5, δ_c_ 40.4, δ_c_ 40.9, δ_c_ 133.6, δ_c_ 128.2, δ_c_ 29.5, δ_c_ 43.6, δ_c_ 70.4, δ_c_ 56.4, δ_c_ 173.6, and δ_c_ 46.0 ppm that were ascribed to C-1 to C-8, and C-13 to C-15, respectively. Moreover, HMBC spectra evidenced and confirmed the amino acid sequence in jaspamide Q (**2**) through cross-peaks between N*H*-Tyr and C-12, Me-17 and C-14, and between N*H*-Ala and C-1.

Jaspamide R (**3**) was isolated as a white amorphous solid. Its ESI mass spectrum exhibited pseudomolecular ion peaks at *m/z* 787.1, 789.1, and 791.1 [M+H]^+^, in a ratio of 1:2:1, supporting the existence of two bromine atoms in the compound. The molecular formula of jaspamide R (**3**) was determined to be C_36_H_44_Br_2_N_4_O_6_ by HRFTMS (*m/z* 789.1691 [M+H]^+^, Δ + 1.0 ppm) which exceeds that of jaspamide (**1**) by 79 amu revealing that jaspamide R (**3**) is a dibromo analogue of jaspamide Q (**2**).

This difference was explained by the ^1^H-NMR spectral data (Table 1), which revealed close similarity between jaspamide R (**3**) and jaspamide (**1**), except for the proton resonances corresponding to the indole moiety of the 2-bromoabrine unit. Jaspamide R (**3**) showed three proton resonances at δ_H_ 7.41 (1H, br *s*), δ_H_ 7.20 (1H, *d*, 8.2 Hz), and δ_H_ 7.42 (1H, *d*, 8.2 Hz) that were assigned to H-21, H-23, and H-24, respectively. Whereas for jaspamide (**1**), four resonances, at δ_H_ 7.23 (1H, *d*, 8.0 Hz), δ_H_ 7.10 (1H, *t*, 8.0 Hz), δ_H_ 7.12 (1H, *t*, 8.0 Hz), and δ_H_ 7.54 (1H, *d*, 8.0 Hz), ascribed for H-21 to H-24, were observed. Based on this finding, the additional bromine atom of **3** was assumed to be located at C-22. This hypothesis was further confirmed by 2D NMR spectral analyses including ^1^H–^1^H COSY, TOCSY, and NOESY spectra that revealed a clear NOE correlation between proton resonance at δ_H_ 7.41 (1H, br *s*), and N*H*-Tyr at δ_H_ 7.53 (1H, *d*, 8.6 Hz) proving the attachment of the second bromine atom to be at C-22. Moreover, the close resemblance in ^1^H resonances between jaspamide R (**3**), and jaspamide (**1**) supports the notion that the chiral centers of alanine, 2,4-dibromoabrine, *β*-tyrosine, and the polypropionate fragment have the same relative configurations in both molecules (Figure 1). Again, due to the lack of material isolated of **3** (0.5 mg), analysis of the absolute configurations of the amino acids, e.g. by Marfey’s method, could not be performed.

To the best of our knowledge, fifteen jaspamide congeners (B–P) were hitherto isolated from the genus *Jaspis* [14,18–21] and all of them show antiproliferative activity with IC_50_ values ranging from 0.01 to 33 *μ*M against human breast adenocarinoma (MCF-7), and colon carcinoma (H-29) cell lines [21].

Jaspamide Q (**2**) and R (**3**) together with the parent jaspamide (**1**) differ in the bromination pattern of the abrine (*N*-methyltryptophan) moiety. Since these modifications were claimed as essential for the observed biological activity [44], compounds (**2** and **3**) together with jaspamide (**1**) were subjected to a cytotoxicity (MTT) assay against mouse lymphoma (L5178Y) cell lines. They exhibited potent activities with IC_50_ values in the ng/mL range (<0.1 *μ*g/mL, <0.16 *μ*M), compared to kahalalide F (IC_50_ = 6.3 *μ*g/mL, 4.3 *μ*M) which was used as a positive control. Further studies aimed at determining the effect of bromination pattern of the abrine residue on cytototoxic activity are in progress.

## 3. Experimental Section

### General experimental procedures

Column chromatography was carried out on Sephadex LH-20 using methanol as an eluent. For analytical HPLC analysis, samples were injected into a HPLC system equipped with a photodiode array detector (Dionex, Munich, Germany). Routine detection was at 235, 254, 280, and 340 nm. The separation column (125 × 4 mm ID) was prefilled with C-18 Eurosphere, 5 μm (Knauer, Berlin, Germany). Separation was achieved by applying a linear gradient from 90% H_2_O (pH 2.0) to 100% MeOH over 40 min. TLC analysis was carried out using aluminium sheet precoated with silica gel 60 F_254_ (Merck, Darmstadt, Germany).

Preparative HPLC separations were performed on a LaChrom-Merck Hitachi HPLC system, pump L-7100, UV detector L-7400 using a C-18 column (Knauer, 300 × 8 mm ID, prefilled with C-18 Eurosphere, flow rate 5 mL/min, UV detection at 280 nm), and the solvent system consisted of a linear gradient of MeOH and nanopure H_2_O.

Optical rotations were measured on a Perkin-Elmer-241 MC polarimeter. ESIMS were obtained on a ThermoFinnigan LCQ DECA mass spectrometer coupled to an Agilent 1100 HPLC system equipped with a photodiode array detector. HRFTMS was recorded on a LTQ FT-MS-Orbitrap (ThermoFinnigan, Bremen, Germany). 1D and 2D NMR spectra were recorded at 300 ºK on a Bruker ARX-500. Samples were dissolved in deuterochloroform.

### Biological material

In August 2008, specimens of *J. splendens* were collected on three neighboring Islands from East Kalimantan (Indonesia), namely Samama, Panjang, and Shoal Islands, at 10 meter depths. Numbers of voucher specimens are RMNH Por. 4234, 4266 and 4299, respectively. They were taxonomically identified as *Jaspis splendens* (order Astrophorida, family Ancorinidae) at the National Museum of Natural History, Leiden, Netherlands. HPLC and LCMS analyses of the three samples revealed that they were identical with regard to their peptide derivatives. Hence, the material was combined in order to obtain sufficient amounts of compounds for subsequent structure elucidation.

### Extraction and isolation

The animal was freeze-dried, and the material (500 g) was extracted with methanol (3 × 2 L) and filtered. The extract was then combined, evaporated to dryness, and partitioned as follows. The methanolic extract (80 g) was dissolved in water and partitioned against *n*-hexane, ethyl acetate, and then *n*-butanol. The bioactive ethyl acetate soluble fraction (2 g) was chromatographed by CC using Sephadex LH20 as stationary phase and eluted with methanol followed by reversed-phase (C18 Eurosphere 100) HPLC using gradient elution of MeOH:H_2_O to yield 105 mg of jaspamide (**1**), 0.7 mg of jaspamide Q (**2**), and 0.5 mg of jaspamide R (**3**).

*Jaspamide Q* (**2**) was obtained as a white amorphous solid: [α]^20^_D_ −62.0° (*c* 0.01, CHCl ); UV (MeOH) λ_max_ 226, 280 nm; ^1^H-NMR see Table 1; ESI-MS pos *m/z* 631.3 [M+H]^+^ (100), ESI-MS neg *m/z* 629.3 [M-H]^−^ (100), HRFTMS *m/z* 631.3491 [M+H]^+^ (calcd for C_36_H_47_N_4_O_6_, 631.3490), and *m/z* 653.3307 [M+Na]^+^ (calcd for C_36_H_46_N_4_O_6_Na, 653.3310).

*Jaspamide R* (**3**) was obtained as a white amorphous solid: [α]^20^_D_ −100.0° (*c* 0.01, CHCl ); UV (MeOH) λ_max_ 231, 283 nm; ^1^H-NMR see Table 1; ESI-MS pos *m/z* 787.1, 789.1, 791.1 [M+H]^+^, 1:2:1; ESI-MS neg *m/z* 785.2, 787.1, 789.0 [M-H] ^−^, 1:2:1; HRFTMS *m/z* 789.1691 [M+H]^+^ (calcd for C_36_H_45_N_4_O_6_^79^Br_2_, 789.1680), and *m/z* 811.1507 [M+Na]^+^ (calcd for C_36_H_44_N_4_O_6_^79^Br_2_Na, 811.1499).

### Cell proliferation assay

Cytotoxicity was tested against L5178Y mouse lymphoma cells using the microculture tetrazolium (MTT) assay as described earlier [11,45]. All experiments were carried out in triplicate and repeated three times. As controls, media with 0.1% EGMME/DMSO were included in the experiments.

## Figures and Tables

**Table 1:** ^1^H-NMR data of jaspamide Q (**2**), and R (**3**), (500 MHz, CDCl_3_-*d*).

Position	Jaspamide Q (2)	Jaspamide R (3)
	δ_H_ mult. (*J* Hz)	δ_H_ mult. (*J* Hz)
2	2.51 (1H, *m*)	2.49 (1H, *m*)
3	A) 2.37 (1H, *dd*, 11.5, 15.8 Hz)	A) 2.37 (1H, *dd*, 11.5, 15.8 Hz)
	B) 1.90 (1H, *d*, 15.8 Hz)	B) 1.90 (1H. *d*, 15.8 Hz)
5	4.79 (1H, *d*, 6.7 Hz)	4.74 (1H, *d*, 6.7 Hz)
6	2.23 (1H, *m*)	2.23 (1H, *m*)
7	A) 1.32 (1H, *m*), B) 1.10 (1H, *m*)	A) 1.32 (1H, *m*), B) 1.10 (1H, *m*)
8	4.64 (1H, *m*)	4.60 (1H, *m*)
10	A) 2.67 (1H, *dd*, 4.7, 15.0 Hz)	A) 2.67 (1H, *dd*, 4.7, 15.0 Hz)
	B) 2.61 (1H, *dd*, 5.7, 14.8 Hz)	B) 2.61 (1H, *dd*, 5.7, 14.8 Hz)
11	5.25 (1H, *dd*, 5.3, 8.6 Hz)	5.25 (1H, *dd*, 5.3, 8.6 Hz)
13	5.63 (1H, *dd*, 6.3, 10.0 Hz)	5.73 (1H, *dd*, 6.3, 10.0 Hz)
15	4.77 (1H, *m*)	4.72 (1H, *m*)
16	1.04 (3H, *d*, 6.6 Hz)	0.81 (3H, *d*, 6.7 Hz)
17	2.97 (3H, *s*)	2.98 (3H, *s*)
18	A) 3.43 (1H, *dd*, 6.3, 15.5 Hz)	A) 3.33 (1H, *dd*, 6.3, 15.5 Hz)
	B) 3.17 (1H, *dd*, 10.4, 15.2 Hz)	B) 3.18 (1H, *dd*, 10.4, 15.2 Hz)
21	7.61 (1H, *d*, 8.0 Hz)	7.41 (1H, br *s*)
22	7.11 (1H, *t*, 8.0 Hz)	
23	7.18 (1H, *t*, 8.0 Hz)	7.20 (1H, *d*, 8.2 Hz)
24	7.35 (1H, *d*, 8.0 Hz)	7.42 (1H, *d*, 8.2 Hz)
26	6.87 (1H, br *s*)	
28	6.90 (1H, *d*, 8.3 Hz)	6.98 (1H, *d*, 8.4 Hz)
29	6.69 (1H, *d*, 8.3 Hz)	6.71 (1H, *d*, 8.4 Hz)
31	6.69 (1H, *d*, 8.3 Hz)	6.71 (1H, *d*, 8.4 Hz)
32	6.90 (1H, *d*, 8.3 Hz)	6.98 (1H, *d*, 8.4 Hz)
33	1.06 (3H, *d*, 6.3 Hz)	1.05 (3H, *d*, 6.1 Hz)
34	0.83 (3H, *d*, 6.5 Hz)	0.81 (3H, *d*, 6.7 Hz)
35	1.59 (3H, *s*)	1.57 (3H, *s*)
36	1.15 (3H, *d*, 6.8 Hz)	1.13 (3H, *d*, 6.8 Hz)
N*H*-Tyr	7.46 (1H, *d*, 8.6 Hz)	7.53 (1H, *d*, 8.6 Hz)
N*H*-Trp	8.24 (1H, br *s*)	8.42 (1H, br *s*)
N*H*-Ala	6.73 (1H, *d*, 6.4 Hz)	6.65 (1H, *d*, 6.5 Hz)
